# Aberrant expression for microRNA is potential crucial factors of haemorrhoid

**DOI:** 10.1186/s41065-020-00139-9

**Published:** 2020-07-03

**Authors:** Chengkun Song, Haikun Zhou, Hong Lu, Chunsheng Luo, Chen Wang, Qingming Wang, Yunhua Peng, Yaojie Xin, Te Liu, Wei Yang

**Affiliations:** 1 Department of Anorectal Surgery, Shuguang Hospital, Shanghai University of Traditional Chinese Medicine, 528 Zhangheng Road, Shanghai, 201203 P.R. China; 2 Department of Otolaryngology, Shuguang Hospital, Shanghai University of Traditional Chinese Medicine, Shanghai, 201203 China; 3 Shanghai Geriatric Institute of Chinese Medicine, Shanghai University of Traditional Chinese Medicine, 365 South Xiangyang Road, Shanghai, 200031 P.R. China; 4 Department of Pathology, Yale University School of Medicine, New Haven, CT 06520 USA

**Keywords:** Haemorrhoids, microRNA, RNA-Seq, Signal transduction pathways

## Abstract

**Background:**

Haemorrhoids occur commonly and frequently in the human digestive system. There are diverse causes of haemorrhoids and their in-depth pathogenesis is still currently unclear.

**Methods:**

In this study, we explored haemorrhoids from an epigenetics perspective by employing RNA-Seq for comprehensive and in-depth analysis of the differences in microRNA (miRNA) transcripts between haemorrhoidal tissue and normal tissue in 48 patients with Grade II and above haemorrhoids.

**Results:**

The results showed that 9 miRNAs were significantly upregulated (ratio > 3.5 and *P*-value < 0.01) and 16 miRNAs were significantly downregulated (ratio > 0.6 and *P*-value < 0.01) in haemorrhoid tissue. Subsequently, target gene prediction results showed that there were 184 potential target genes of significantly upregulated miRNAs (common to both TargetScan7.1 and MirdbV5 databases) and there were 372 potential target genes of significantly downregulated miRNAs. Gene ontology analysis results showed that the target genes of differentially expressed miRNAs in haemorrhoids are involved in regulating “cell composition” and “protein binding”. Lastly, KEGG search found that the differentially expressed miRNAs that are associated with the occurrence of haemorrhoids mainly regulate the activity of endocytosis and the synaptic vesicle cycle.

**Conclusions:**

In summary, the results of high-throughput RNA-Seq screening suggested that the occurrence of haemorrhoids may be intimately associated with aberrant miRNA transcription, resulting in aberrant target gene expression and an imbalance in certain signal transduction pathways.

## Introduction

Piles, also known as haemorrhoids, is a common disease of the anus. This condition can occur at any age but its incidence increases with age [1, 2]. The main presentation of haemorrhoids is haematochezia, characteristics of which include painless, intermittent, fresh blood after bowel movements, dripping during bowel movements or blood on toilet paper, constipation, or aggravation after alcohol consumption or consumption of irritant foods. The location where haemorrhoids occur can be classified as internal haemorrhoids, external haemorrhoids, or mixed haemorrhoids [1–3]. The aetiology of haemorrhoids is extremely complex, yet their incidence is extremely high. Personal hygiene, lifestyle habits, dietary habits, physical differences, genetic factors, and immune factors can all induce the formation of haemorrhoids [4–6]. However, there is a lack of in-depth and comprehensive research on the role of epigenetic regulation in the development of haemorrhoids.

Epigenetics research has found that a class of non-coding RNAs are present in the cytoplasm with lengths of 21–23 nt. These non-coding RNAs are highly conserved during species evolution and in sequence homology. However, there are no open reading frames (ORF) in these sequences so they do not encode any polypeptides. These signs suggest that they play important physiological roles in cells and biological organisms [7–12]. In our previous study, we found that DLK1-DIO3 Imprinted Cluster miRNA expression differed between clinical haemorrhoid samples and normal perianal tissues. An in-depth study found that miRNA-412-5p regulates the epigenetic mechanism of Exportin1 (Xpo1) and down stream p53/p66Shc/p16 pathway on vascular endothelial cell proliferation and haemorrhoid formation [13]. Therefore, the microRNA regulation for the occurrence of hemorrhoids is very important. However, there have been no in-depth and comprehensive reports on whether miRNAs play regulatory roles during the occurrence and development of haemorrhoids. Therefore, this paper uses human haemorrhoidal tissue as study subjects. Transcriptomics methods (RNA-Seq) was employed as a study method with healthy human tissue as controls for comprehensive analysis of differentially expressed miRNA transcript population. In addition, bioinformatics was used to examine the potential target genes of the aforementioned miRNAs and their functions.

## Materials and methods

### Collection and grouping of tissue samples

In this study, haemorrhoidal samples were collected from 48 male patients who underwent haemorrhoid surgery in the Department of Proctology in Shanghai Shuguang Hospital from September 2017 to April 2018. The ages of all patients were between 28 to 50 years (40 ± 8), and the clinical stage of haemorrhoids was Grade III or IV. The study protocol was approved by the Regional Ethics Committee of Shuguang Hospital, Shanghai University of Traditional Chinese Medicine (Permission No.: 201701.4), in accordance with the 2008 Helsinki declaration. Written informed consent was obtained from every patient and control.

### RNA extraction

RNA was extracted using the RNAprep pure Tissue Kit (TIANGEN Biotech (Beijing) Co., Ltd), following the manufacturer’s instructions. Briefly, 20 mg of human tissue samples were taken and 800 μl of lysis buffer was added before homogenisation. The supernatant was obtained and 200 μl of chloroform was added and mixed evenly by inverting. Following that, the samples were centrifuged at 13,400×*g* for 15 min at 4 °C and the supernatant was collected. Two volumes of absolute ethanol were added to the supernatant and mixed evenly. Following that, the samples were centrifuged at 13,400×*g* for 30 min at 4 °C. Five hundred microlitersof 75% ethanol was added to the RNA precipitate for resuspension before centrifugation at 13,400×*g* for 5 min at 4 °C. Excess liquid was removed and 300 μl DEPC water was added to solubilise the precipitate. RNA solution (1 μl) was used to measure the OD_260_/OD_280_ (generally controlled within 1.8–2.0) to determine the purity and total concentration of RNA.

### Reverse transcription of RNA into cDNA

Reverse transcription was carried out using the miRcute miRNA First-strand cDNA (TIANGEN Biotech (Beijing) Co., Ltd) kit, following the manufacturer’s instructions. Briefly, 20 μl (100 ng/μl) of total RNA, 25 μl of 2 × miRNA RT Reaction Buffer, 4 μl of 1 × miRNA RT Enzyme Mix and 6 μl of RNAse-free deionised water were fully mixed. The following reaction was carried out on a PCR machine: 42 °C for 60 min for the addition of a poly (A) tail to miRNA and reverse transcription followed by 95 °C for 3 min for enzyme inactivation.

### miRNA qPCR

qPCR was performed using the miRcute miRNA qPCR Detection (TIANGEN Biotech (Beijing) Co., Ltd) kit, following the manufacturer’s instructions. Briefly, the following reaction system was set up: 10 μl of 2 × miRcute Plus miRNA Premix (with SYBR), 1 μl (10 μM) each of 1 × Forward Primer and Reverse Primer, 4 μl of miRNA first strand cDNA, and 4 μl of deionised water. The following reaction was carried out on areal-time fluorescence quantitative PCR machine: 95 °C for 15 min, 94 °C for 20 s, 60 °C for 34 s, and the fluorescence value was read. The aforementioned reaction was repeated for 40 cycles. Table 1 shows the qPCR primers.

**Table 1:** qPCR primers

Gene Name	Forward (F) and reverse (R) primers (5’ → 3’)
hsa-miR-375	F: TTTGTTCGTTCGGCTCGCGTGA R: GCTGTCAACGATACGCTACCTA
hsa-miR-215-5p	F: ATGACCTATGAATTGACAGAC R: GCTGTCAACGATACGCTACCTA
hsa-miR-192-5p	F: CTGACCTATGAATTGACAGCC R: GCTGTCAACGATACGCTACCTA
hsa-miR-143-3p	F: TGAGATGAAGCACTGTAGCTC R: GCTGTCAACGATACGCTACCTA
hsa-miR-187-3p	F: TCGTGTCTTGTGTTGCAGCCGG R: GCTGTCAACGATACGCTACCTA
hsa-miR-194-5p	F: TGTAACAGCAACTCCATGTGGA R: GCTGTCAACGATACGCTACCTA
hsa-miR-194-5p	F: TGTAACAGCAACTCCATGTGGA R: GCTGTCAACGATACGCTACCTA
hsa-miR-145-5p	F: GTCCAGTTTTCCCAGGAATCCCT R: GCTGTCAACGATACGCTACCTA
hsa-miR-490-3p	F: CAACCTGGAGGACTCCATGCTG R: GCTGTCAACGATACGCTACCTA
hsa-miR-145-3p	F: GGATTCCTGGAAATACTGTTCT R: GCTGTCAACGATACGCTACCTA
hsa-miR-139-3p	F: TGGAGACGCGGCCCTGTTGGAGT R: GCTGTCAACGATACGCTACCTA
hsa-miR-376b-3p	F: ATCATAGAGGAAAATCCATGTT R: GCTGTCAACGATACGCTACCTA
hsa-miR-34a-5p	F: TGGCAGTGTCTTAGCTGGTTGT R: GCTGTCAACGATACGCTACCTA
hsa-miR-379-3p	F: TATGTAACATGGTCCACTAACT R: GCTGTCAACGATACGCTACCTA
hsa-miR-190b	F: TGATATGTTTGATATTGGGTT R: GCTGTCAACGATACGCTACCTA
hsa-miR-152-3p	F: TCAGTGCATGACAGAACTTGG R: GCTGTCAACGATACGCTACCTA
hsa-let-7c-5p	F: TGAGGTAGTAGGTTGTATGGTT R: GCTGTCAACGATACGCTACCTA
hsa-miR-196b-5p	F: TAGGTAGTTTCCTGTTGTTGGG R: GCTGTCAACGATACGCTACCTA
hsa-miR-107 hsa-miR-517a-3p	F: ATCGTGCATCCCTTTAGAGTGT R: GCTGTCAACGATACGCTACCTA
hsa-miR-517b-3p	F: ATCGTGCATCCCTTTAGAGTGT R: GCTGTCAACGATACGCTACCTA
hsa-miR-1307-5p	F: TCGACCGGACCTCGACCGGCT R: GCTGTCAACGATACGCTACCTA
18S rRNA	F: CAGCCACCCGAGATTGAGCA R: TAGTAGCGACGGGCGGTGTG
CLTA	F: TCCAACAGACAGTTATGCAGC R: CCATTTACGGATACTTTCAGGCT
CLTC	F: ATTCTGCCAATTCGTTTTCAGGA R: GCTTTCAGTGCAATTACTTTGCT
RAB3A	F: GAGTCCTCGGATCAGAACTTCG R: TGTCGTTGCGATAGATGGTCT
SNAP25	F: TCGTGTAGTGGACGAACGG R: TCTCATTGCCCATATCCAGGG
SYT1	F: GTGAGCGAGAGTCACCATGAG R: CCCACGGTGGCAATGGAAT
VAMP2	F: CTCAAGCGCAAATACTGGTGG R: TGATGGCGCAAATCACTCCC
AP2B1	F: ATCCAATGGTGGTGGCTAATG R: TGGGTTCAGATCAAGTAAGTTGC
ARF6	F: GGGAAGGTGCTATCCAAAATCTT R: CACATCCCATACGTTGAACTTGA
ASAP3	F: ACGCCCAGCACAACTTTTTC R: GGCTCAGGTGTTCCTCTCT
DAB2	F: GTAGAAACAAGTGCAACCAATGG R: GCCTTTGAACCTTGCTAAGAGA
PSD3	F: GGAGAAAGCTAACGGAACACA R: TGAGGAATGTCCAAAAATGGGTT
RAB11FIP1	F: AGAACAGCGAGTACGGATCTT R: GACATCATTCTTAGACCGAAGGC
STAM	F: AGGAGCATGTGTATCAAACTGTG R: TGTCCATTCAACCATAAGAGCC
TRAF6	F: TTTGCTCTTATGGATTGTCCCC R: CATTGATGCAGCACAGTTGTC

### Haematoxylin-eosin (H&E) staining

Tissue samples were fixed with 4% paraformaldehyde followed by dehydration and paraffin embedding. Four-micronthick sections were cut from the paraffin blocks and pasted on glass slides. Subsequently, xylene was used for clearing followed by dehydration with an ethanol gradient (100, 90, 80%) dehydration. Haematoxylin staining was carried out at room temperature for 5 min before differentiation using 1% hydrochloric acid in ethanol, bluing with ammonia water for 1 min, and rinsing with distilled water for 5 min. Subsequently, eosin staining at room temperature for 2 min was carried out followed by rinsing with distilled water for 2 min. This was followed by ethanol gradient (75, 80, 95, 100%) decolorisation. Xylene was used for clearing for 2 min. Finally, neutral resin was used for mounting.

### RNA-Seq

Construction of miRNA libraries and RNA-Seq was completed by KangChen Bio-tech (Shanghai, China). The haemorrhoidal tissues of 3 patients were used as the sample group and subcutaneous scar tissue of healthy people wasused as the normal control group. The miRNA libraries were constructed following the manufacturer’s protocol. Briefly, the total RNA was extracted from samples in each group to prepare the miRNA sequencing libraries. This includes the following steps: 1) 3′ adapter ligation; 2) 5′ adapter ligation; 3) cDNA synthesis; 4) PCR amplification; 5) Selection of 135–155 bp PCR amplicons (correspond to 15–35 nt small RNAs). Libraries were denatured into single-strand DNA molecules and captured using the Illumina flow cell. This was followed by in situ amplification into clusters. Further, 51 sequencing cycles were carried out on anIllumina NextSeq 500 sequencer, following the manufacturer’s instructions.

### Data analysis and statistical methods

Solexa CHASTITY was used for quality control and screening of raw reads from data obtained from RNA-Seq to obtain clean reads. Adapters were removed from clean reads to obtain trimmed reads with a tag of ≥15 nt. The miRDeep2 software was used for prediction of new miRNAs from all trimmed reads to obtain new pre-miRNAs. Following that, the Novoalign software (v2.07.11) was used to align trimmed reads to the merged pre-miRNA database (miRBase v21 pre-miRNAs + newly predicted pre-miRNAs) with at most 1 mismatch allowed. During calculation of miRNA expression, reads with a quantity less than 2 were discarded. In order to characterise changes in isoforms, ±4 nt reads in mature forms were considered as isoforms of mature miRNAs and classified as 5P and 3P species according to their position on the precursor hairpin. The number of reads in every mature miRNA region (±4 nt) was summed as the raw expression level and tag counts per million miRNA alignments (TPM) was used for standardizing samples. We calculated the expression levels of mature miRNAs, isoforms with the highest expression level, and all miRNA isoforms to satisfy the different requirements of customers. The number of tags on all standardised miRNA isoforms was used to calculate the *P*-value and fold change of the two groups of samples (with replicates) and these values were used to screen for differentially expressed miRNAs. The fold change between two samples (without replicates) was calculated to screen for differentially expressed miRNAs. A cluster map of miRNAs was plotted. A miRNA target gene prediction software was used to predict the target genes of top 10 differentially expressed miRNAs and these target genes were used for Gene Ontology (GO) and Pathway analysis.

## Results

### Significant differences in miRNA expression exist between haemorrhoidal tissue and healthy tissue

**Fig. 1:**
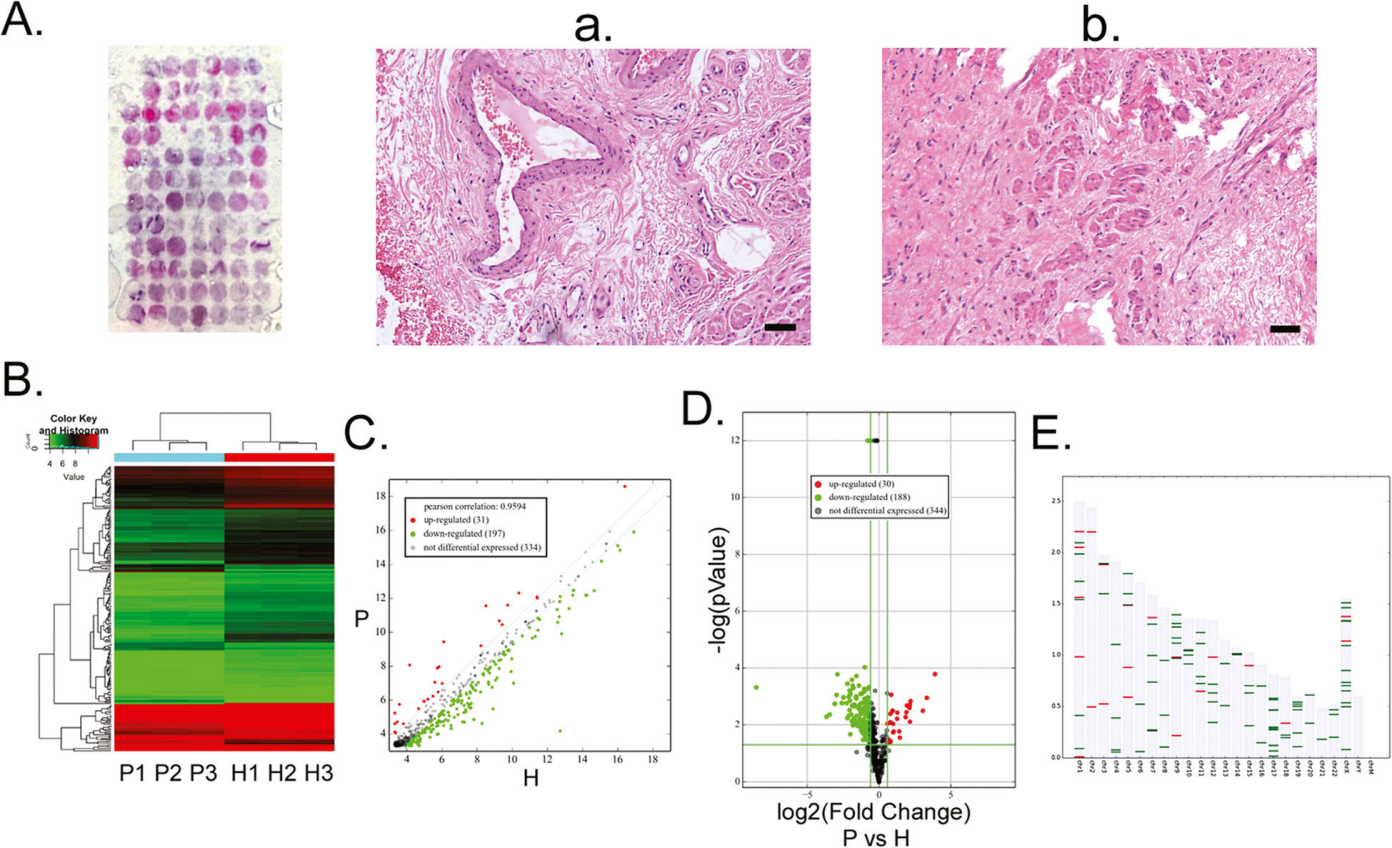
Significant microRNA (miRNA) expression differences exist between haemorrhoidal tissue and healthy tissue. **a** Haematoxylin & eosin (HE) staining results of tissue microarrays of haemorrhoidal tissue and healthy tissue from patients. 40× magnification. (**a**) HE staining results of haemorrhoidal tissue. 200× magnification. Scale bar = 40 μm. **b** HE staining results of healthy tissue. 200× magnification. Scale bar = 40 μm. **b** Cluster search results of differentially expressed miRNAs between haemorrhoidal and healthy tissue from RNA-Seq analysis. P: haemorrhoid patient samples; H: healthy tissue samples. **c** Scatter plot of differentially expressed miRNAs between haemorrhoidal and healthy tissue from RNA-Seq analysis. P: haemorrhoid patient samples; H: healthy tissue samples. **d** Volcano plot of differentially expressed miRNAs between haemorrhoidal and healthy tissue from RNA-Seq analysis. P: haemorrhoid patient samples; H: healthy tissue samples. (E) Chromosomal locations of differentially expressed miRNAs between haemorrhoidal and healthy tissue from RNA-Seq analysis

Before transcriptome sequencing of miRNAs was carried out, we carried out pathological staining of all collected tissue samples to determine their pathological characteristics and staging. From the H&E staining results, several tortuous and dilated veins couldbe observedat the layer below the haemorrhoidal mucosa, with thick walls surrounding elastic fibres. Intraluminal thrombi were present in individual tissues. Interstitial oedema was apparent and diffuse linear and wavy elastic fibres were observed between collagen fibres. Many dilated and distended veins were present in the mucosa of haemorrhoids with small amounts of erythrocytes and inflammatory cells (Fig. 1). Compared with the pathological characteristics of haemorrhoidal tissue, no thrombus, oedema, or inflammatory cells were observed in healthy tissue (Fig. 1). The reads obtained from RNA-Seq were aligned to pre-miRNAs to calculate the number of miRNA alignments as the expression level of miRNA. The miRNA expression information of mature miRNA, most abundant isomiRNA, and all isoforms were calculated and TPM (number of reads of aligned miRNA *1 million/aligned reads) was used for standardisation. Finally, all miRNA isoform data was used to calculate the differentially expressed miRNAs between samples. We obtained differential miRNA transcriptome data between haemorrhoidal and healthy tissues that are more complete and comprehensive through bioinformatics analysis. These results showed that 1942 miRNA molecules were obtained from sequencing (Fig. 1). These miRNAs are distributed in chromosomes other than the Y chromosome (Fig. 1). In these miRNAs, 9 miRNAs were significantly upregulated (ratio > 3.5 and *P*-value < 0.01) and 16 miRNAs were significantly downregulated (ratio > 0.6 and *P*-value < 0.01) (Table 2). The results showed that many differentially expressed miRNAs were present between haemorrhoidal and healthy tissues.

**Table 2:** Restults of microRNA RAN-Seq

Access Number	microRNA	Mature Microrna Length	Mature microRNA Sequence	P vs H Fold Change	***P***-value	State
MIMAT0000728	miR-375	22	UUUGUUCGUUCGGCUCGCGUGA	14.944	< 0.000	up
MIMAT0000272	miR-215-5p	21	AUGACCUAUGAAUUGACAGAC	10.072	0.001	up
MIMAT0000222	miR-192-5p	21	CUGACCUAUGAAUUGACAGCC	8.330	0.003	up
MIMAT0000435	miR-143-3p	21	UGAGAUGAAGCACUGUAGCUC	4.600	0.001	up
MIMAT0000262	miR-187-3p	22	UCGUGUCUUGUGUUGCAGCCGG	4.472	0.008	up
MIMAT0000460	miR-194-5p	22	UGUAACAGCAACUCCAUGUGGA	4.450	0.002	up
MIMAT0000437	miR-145-5p	23	GUCCAGUUUUCCCAGGAAUCCCU	3.852	0.002	up
MIMAT0002806	miR-490-3p	22	CAACCUGGAGGACUCCAUGCUG	3.767	0.004	up
MIMAT0004601	miR-145-3p	22	GGAUUCCUGGAAAUACUGUUCU	3.625	0.005	up
MIMAT0002172	miR-376b-3p	22	AUCAUAGAGGAAAAUCCAUGUU	0.667	< 0.000	down
MIMAT0000255	miR-34a-5p	22	UGGCAGUGUCUUAGCUGGUUGU	0.663	0.002	down
MIMAT0000438	miR-152-3p	21	UCAGUGCAUGACAGAACUUGG	0.657	0.003	down
MIMAT0000064	let-7c-5p	22	UGAGGUAGUAGGUUGUAUGGUU	0.657	0.007	down
MIMAT0000104	miR-107	23	AGCAGCAUUGUACAGGGCUAUCA	0.655	0.008	down
MIMAT0002852	miR-517a-3p	22	AUCGUGCAUCCCUUUAGAGUGU	0.652	0.004	down
MIMAT0002857	miR-517b-3p	22	AUCGUGCAUCCCUUUAGAGUGU	0.652	0.004	down
MIMAT0022727	miR-1307-5p	21	UCGACCGGACCUCGACCGGCU	0.650	0.007	down
MIMAT0000458	miR-190a-5p	22	UGAUAUGUUUGAUAUAUUAGGU	0.648	0.004	down
MIMAT0000731	miR-378a-5p	22	CUCCUGACUCCAGGUCCUGUGU	0.645	0.000	down
MIMAT0004927	miR-708-3p	22	CAACUAGACUGUGAGCUUCUAG	0.635	0.006	down
MIMAT0001545	miR-450a-5p	22	UUUUGCGAUGUGUUCCUAAUAU	0.632	0.002	down
MIMAT0001545	miR-450a-5p	22	UUUUGCGAUGUGUUCCUAAUAU	0.623	0.006	down
MIMAT0000692	miR-30e-5p	22	UGUAAACAUCCUUGACUGGAAG	0.617	0.008	down
MIMAT0002888	miR-532-5p	22	CAUGCCUUGAGUGUAGGACCGU	0.616	0.000	down

### Differentially expressed miRNAs in haemorrhoids have many potential target genes

**Fig. 2:**
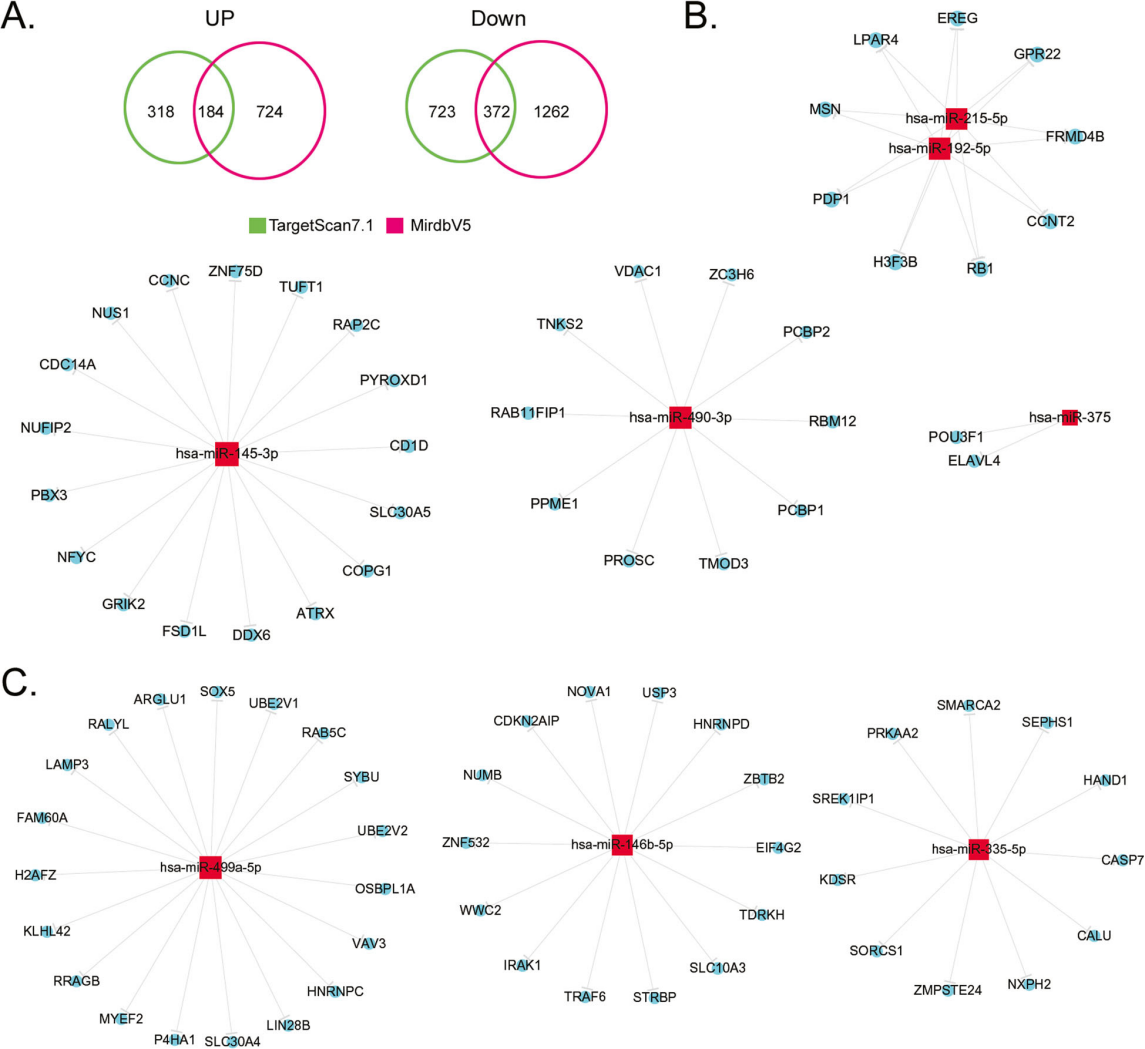
Differentially expressed microRNAs (miRNAs) in haemorrhoids have many potential target genes. **a** Target gene prediction results showed that there are 184 potential target genes of significantly upregulated miRNAs (common to both TargetScan7.1 and MirdbV5 databases) and there are 372 potential target genes of significantly downregulated miRNAs (common to both TargetScan7.1 and MirdbV5 databases). **b** show corresponding potential target genes of significantly upregulated miRNAs. **c** show corresponding potential target genes of significantly downregulated miRNAs

miRNAs are the most abundant class of small RNAs in mammals. They can induce mRNA degradation or inhibit transcription to play important roles in regulating gene expression. Each miRNA binds to the 3′ UTR of target genes by partial sequence complementation. On average, each miRNA has a few hundred target genes. After obtaining the differentially expressed miRNAs from screening, authoritative miRNA databases (TargetScan7.1 and MirdbV5) were used for alignment. Biostatistical calculations were used to screen for potential target genes of the aforementioned miRNAs to construct miRNA-target genes regulatory networks. This was used to deduce the function of these miRNAs. In this study, we predicted the target genes of 7 significantly upregulated miRNAs and 8 significantly downregulated miRNAs (Fig. 2). Bioinformatics analysis showed that the quantities of corresponding target genes of the aforementioned miRNAs differed in different databases. For upregulated miRNAs, 502 and 908 potential target genes were screened from the TargetScan7.1 and MirdbV5 databases, respectively, of which 184 target genes were common to both databases (Fig. 2, Supplementary data). For downregulated miRNAs, 1095 and 1634 potential target genes were screened from the TargetScan7.1 and MirdbV5 databases, respectively, of which 372 target genes were common to both databases (Fig. 2, Supplementary data). We noticed that except for miRNA-133b and miRNA-133a-3p, which had 32 common potential target gene mRNAs, the predicted potential target genes of the remaining 13 miRNAs were all independent genes and there was no overlap (Fig. 2, Supplementary data). The experimental results showed that the aforementioned miRNAs with significant differential expression regulates a large number of potential target genes.

### Gene ontology analysis shows that haemorrhoids contain many physiological and biochemical abnormalities

**Fig. 3:**
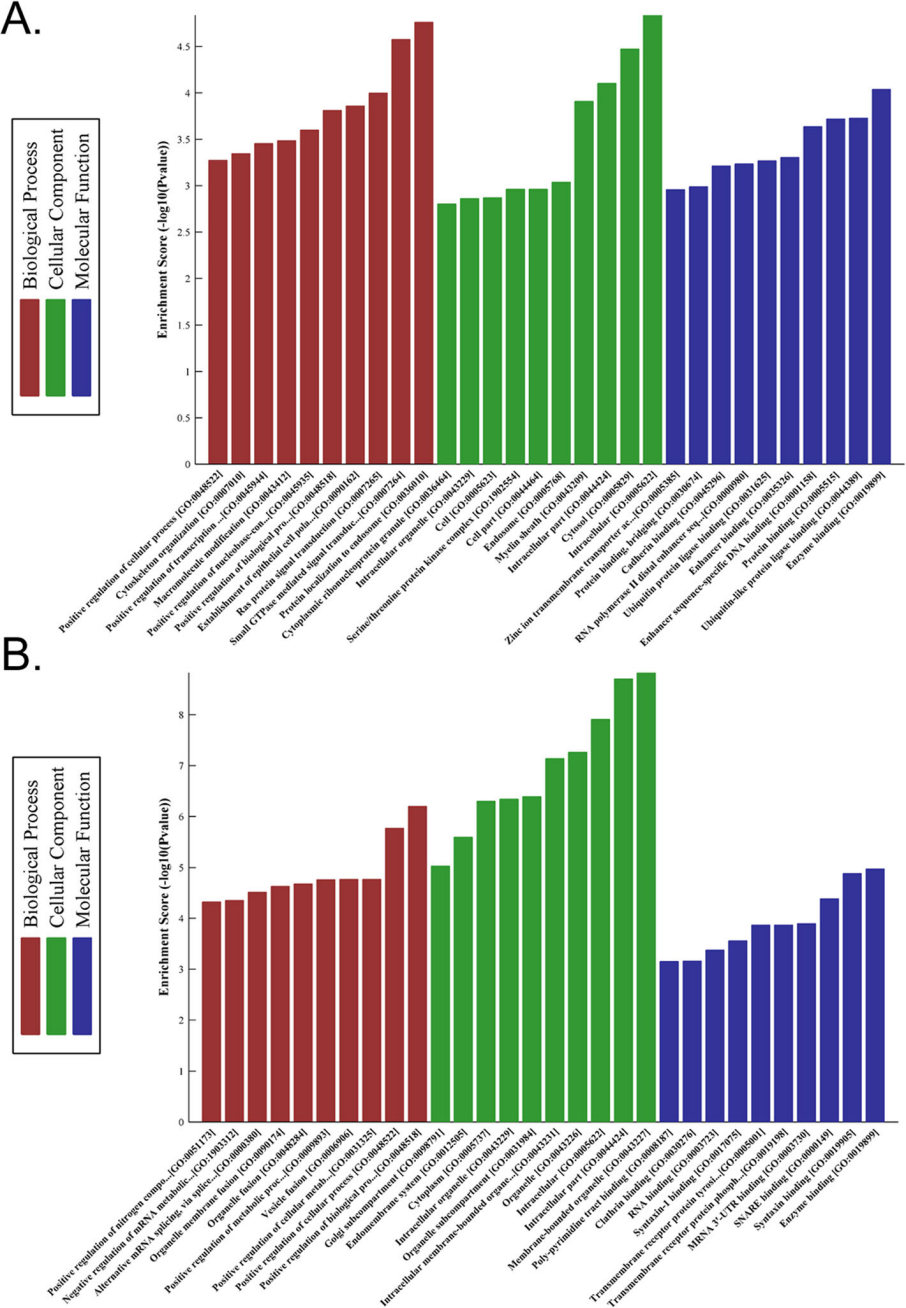
Gene ontology analysis shows that haemorrhoids contain many physiological and biochemical abnormalities. **a** The target genes of upregulated miRNAs are mainly involved in biological regulation, cell part, and protein binding. **b** The target genes of downregulated miRNAs are mainly involved in biological regulation, cell part, and protein binding

GO is an international standard classification standard for gene function and is divided into molecular function (MF), biological process (BP), and cellular component (CC). We employed GO to analyse and deduce the molecular functions of the target genes of the 10 miRNAs with the most significant differential expression to predict the function of these differentially expressed miRNAs. The experimental results showed that the target genes of upregulated miRNAs are mainly involved in BP, CC, and MF (Fig. 3, Supplementary data). The target genes of downregulated miRNAs were mainly involved in BP, CC, and MF (Fig. 3, Supplementary data). GO analysis results showed that the target genes of differentially expressed miRNAs in haemorrhoids mainly regulate cell composition and protein binding.

### Endocytosis and synaptic vesicle cycle are possible regulatory targets of miRNAs during the development of haemorrhoids

**Fig. 4:**
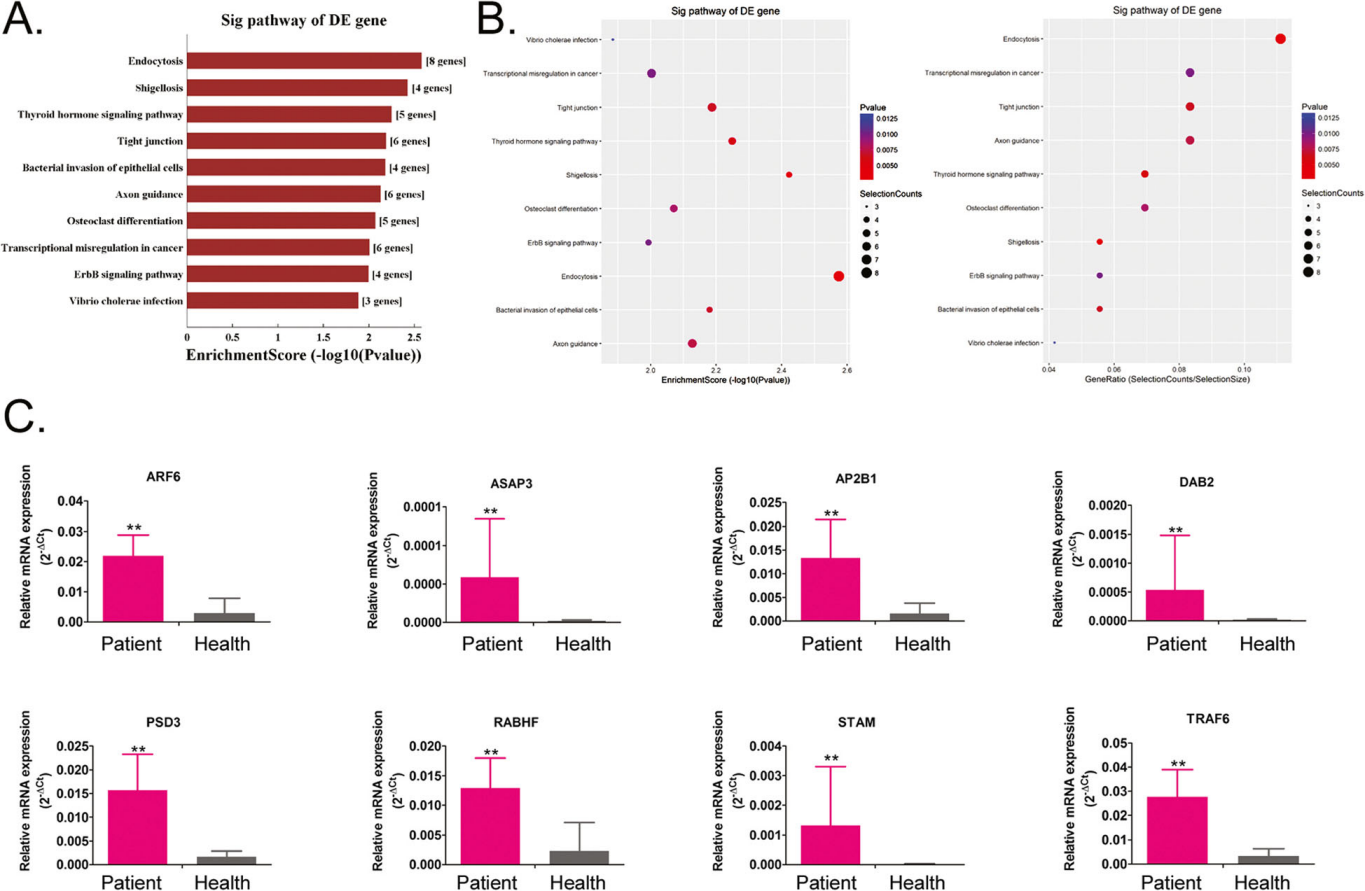
Prediction of target pathways of upregulated microRNAs (miRNAs). **a** and **b** Most target genes of the upregulated miRNAs belong to the endocytosis signalling pathway. **c** The qPCR validation of the expression levels of important node genes on endocytosis signalling pathway. ** *p* < 0.01 vs Healthy tissue samples; *n* = 13; t test

**Fig. 5:**
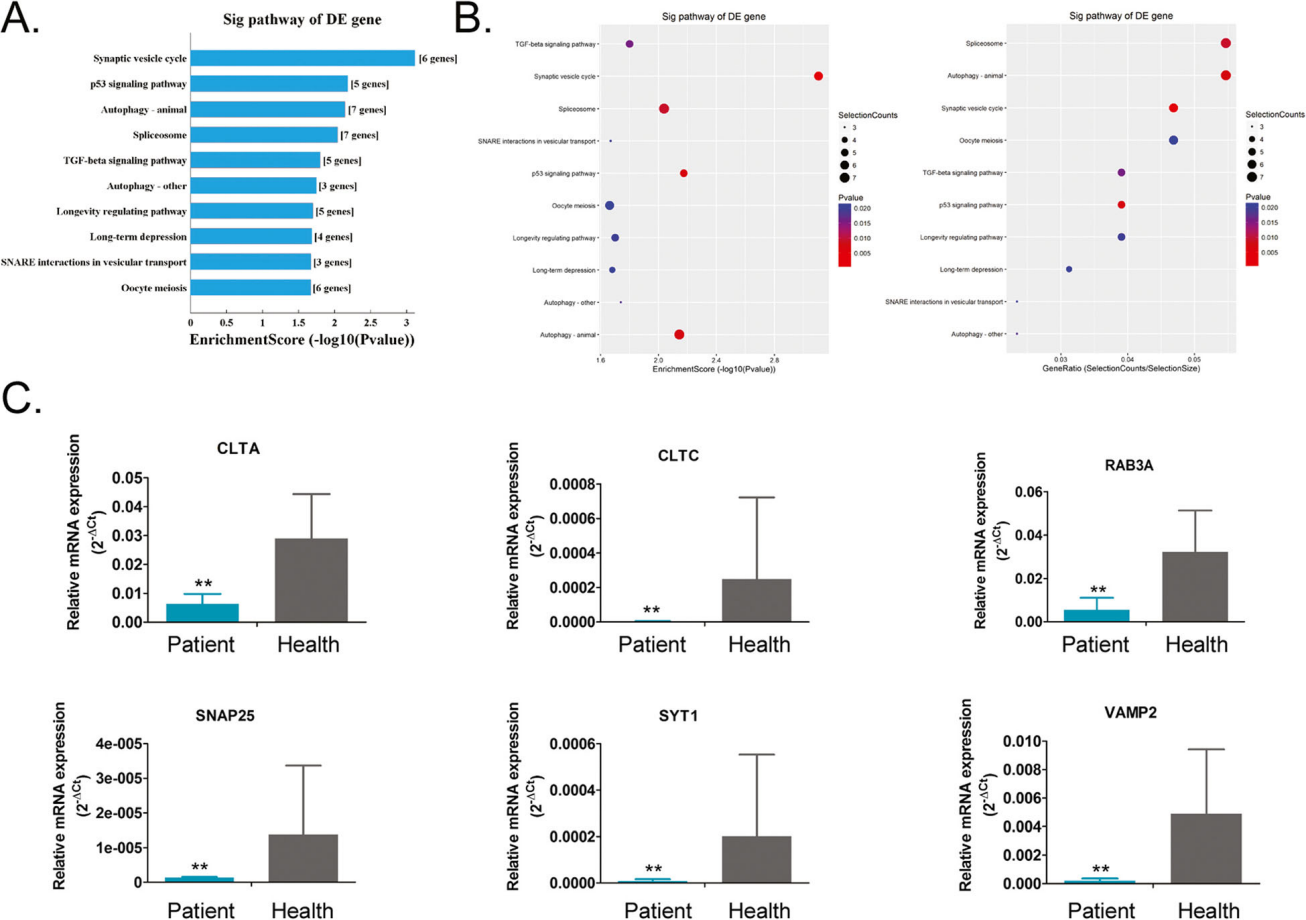
Prediction of target pathways of downregulated microRNAs (miRNAs). **a** and **b** Most target genes of the downregulated miRNAs belong to the synaptic vesicle cycle signalling pathway. **c** The qPCR validation of the expression levels of important node genes on synaptic vesicle cycle signalling pathway. ** *p* < 0.01 vs Healthy tissue samples; *n* = 13; t test

Through RNA-Seq, we screened out differentially expressed miRNAs between haemorrhoidal and healthy tissues. We obtained several potential target genes that are regulated by these miRNAs through alignment with target gene databases. The target genes obtained were aligned with the KEGG database (http://www.genome.jp/kegg/) to identify the biological signal transduction pathways wherethe genes are located. The alignment data showed that the gene populations that are regulated by upregulated miRNAs belong to 10 signalling pathways, of which the endocytosis pathway contained the greatest number of differentially expressed target genes (Fig. 4, Supplementary data). On the contrary, the gene populations that are regulated by downregulated miRNAs belong to 10 signalling pathways, of which the synaptic vesicle cycle pathway is the pathway that contained the greatest number of differentially expressed target genes (Fig. 5, Supplementary data). Meanwhile, the expression levels of important node genes on endocytosis or synaptic vesicle cycle signalling pathway were validated by qPCR. The results showed that the expressioon levels of all important node genes (AP2B1, ARF6, ASAP3, DAB2, PSD3, RAB11FIP1, STAM, TRAF6) on endocytosis pathways from haemorrhoid patient samples were higher significantly than them from healthy tissue samples (Fig. 4). However, the results showed that the expression levels of all important node genes (CLTA, CLTC, RAB3A, SNAP25, SYT1, VAMP2) on Synaptic vesicle cycle pathways from haemorrhoid patient samples were lower significantly than them from healthy tissue samples (Fig. 4). Therefore, the above alignment data shows that the development of haemorrhoids may be associated with imbalances in target genes in the endocytosis and synaptic vesicle cycle pathways that are regulated by miRNAs.

### RNA-Seq high-throughput sequencing results are generally consistent with qPCR validation results

**Fig. 6:**
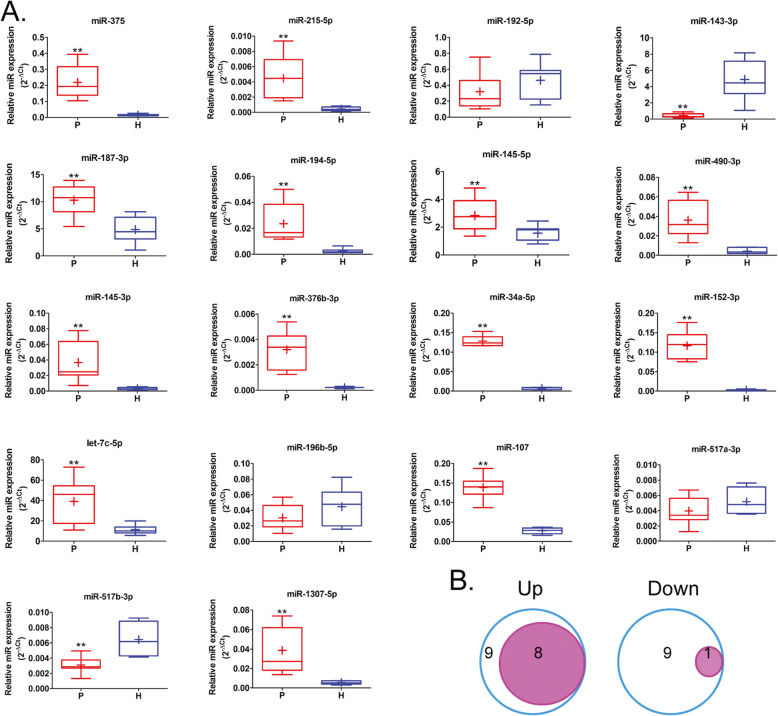
qPCR validation of the accuracy of RNA-Seq results. **a** qPCR validation results. P: haemorrhoid patient samples; H: healthy tissue samples.** *p* < 0.01 vs H; *n* = 10; t test. **b** There were 9 upregulated microRNAs (miRNAs) that matched the RNA-Seq results while only 1 downregulated miRNA matches the RNA-Seq result. The remaining miRNAs did not match the RNA-Seq results

Through data screening, we found that 24 miRNAs showed significant transcript differences between the two groups. From this, we can see that the development of haemorrhoids and differential miRNA expression showed a continuum. From further miRNA-specific qPCR validation, we found that only 1 upregulated miRNA (miR-192-5p) did not match the RNA-Seq results, while the remaining miRNAs matched the RNA-Seq results. For downregulated miRNAs, only 1 upregulated miRNA (miR-517-5p) matched the RNA-Seq results, while the remaining miRNAs did not match the RNA-Seq results (Fig. 6). Therefore, we hypothesised that RNA-Seq has unique advantages in high-throughput screening of differentially expressed transcripts, but there are some errors with the results obtained.

## Discussion

In China, haemorrhoids are a common disease in proctology [1–6, 14]. Haemorrhoids cause much inconvenience in the lives of afflicted people and significantly affect physical and mental health. Generally, internal haemorrhoids can be classified into 4 grades [1–6, 14] --Grade I: Bleeding during bowel movements which spontaneously stop after bowel movement. These haemorrhoids do not prolapse; Grade II: Frequent haematochezia, protrudes out during bowel movement but spontaneously return to the original position; Grade III: Require manual pushing after prolapse; and Grade IV: Permanently prolapsed and cannot be pushed back into position [1–6, 14]. Among these haemorrhoids, Grade II and above haemorrhoids mostly form mixed haemorrhoids, which show both symptoms of internal and external haemorrhoids. Pain and itching may be present. Itching is often caused by viscous secretions during haemorrhoid prolapse [1–6, 14]. The aetiology of haemorrhoids is extremely complex. Currently, there are two relatively recognised theories. The first theory is the varicose vein theory which asserts that haemorrhoids are a mass of veins formed from the congestion, dilatation, and flexion of the venous plexus at the lower mucosa of the rectum and below the skin of the anal canal. In addition, Thomson’s sliding anal canal lining theory has become more and more accepted [1–6, 14]. This theory asserts that haemorrhoids are originally normal anatomical structures in the anal canal, i.e., vascular cushions, which are ring-like spongy tissues at the pectinate line and 1.5 cm above the pectinate line [1–6, 14]. Only when abnormalities in the anal cushion occur along with comorbid symptoms can they be termed as haemorrhoids and require treatment. The objective of treatment is to relieve symptoms and not to eliminate haemorrhoids [1, 3–6, 14]. There are many causes of haemorrhoids, such as constipation, long-term alcohol consumption, constipation of large amounts of food irritants and prolonged sitting are major causes [1–6, 14]. However, the development of haemorrhoids is considered to be intimately associated with lifestyle habits and environmental factors [1–6, 14]. On the contrary, many reports foundthat environmental factors and lifestyle habits can significantly affect epigenetic status and induce diseases. Based on the aforementioned clues, we hypothesised that the development of haemorrhoids should have unique epigenetic variation patterns. There are not many studies in the field of epigenetic regulation on the regulatory mechanisms of non-coding RNAs, particularly miRNAs, on the development of haemorrhoids.

In our study, we focused on miRNAs. By employing RNA-Seq, we conducted a comprehensive and in-depth analysis of the differences in miRNA transcriptomics between haemorrhoidal and healthy tissues and the status of potential target genes that were regulated by these miRNAs. The RNA-Seq results showed that there are transcript differences in thousands of miRNAs between haemorrhoidal and healthy tissues, and data screening showed that 24 of these miRNAs were significantly differentially expressed between the two groups. From this, we can see that the development of haemorrhoids and differential miRNA expression shows a continuum. From further miRNA-specific qPCR validation, we found that only 1 upregulated miRNA did not match the RNA-Seq results, while the remaining miRNAs matched the RNA-Seq results. For downregulated miRNAs, only 1 upregulated miRNA matched the RNA-Seq results while the remaining miRNAs did not match the RNA-Seq results. Therefore, we hypothesised that although RNA-Seq has unique advantages in high-throughput screening of differentially expressed transcripts, there are some errors inthe results obtained. There is still a need for large numbers of clinical samples for qPCR validation of the differential transcriptome data obtained from RNA-Seq in order to identify data that are consistent with the results and remove contradictory data. Subsequently, we identified potential target genes of differentially expressed miRNAs from 2 public databases. The statistical analysis showed that there were several potential target genes, regardless of whether the miRNA was upregulated or downregulated. In addition, there are overlaps in the target genes that are regulated by some miRNAs. These results suggest that the development of haemorrhoids is due to a large and complex miRNA-gene regulatory network. Changes in the expression of some miRNAs will change the expression of a group of target genes. By further classification of the potent target genes that are regulated by the miRNAs obtained from screening, we found that the endocytosis and synaptic vesicle cycle signal transduction pathways are signalling pathways that contain the most target genes. Therefore, we hypothesised that the development of haemorrhoids may be associated with imbalances in target genes in the endocytosis and synaptic vesicle cycle pathways that are regulated by miRNAs.

Aberrant responses to physiological or non-physiological stress signals by the body are usually the cause of disease. The occurrence of haemorrhoids is a good example. This is because there are many causes of haemorrhoids, such as constipation, long-term alcohol consumption, consumptionof large amounts of food irritants, and prolonged sitting. These factors can result in long-term and frequent stimulation of the perianal and rectal regions and be considered as a stable and persistent external stress signal. The occurrence of haemorrhoids is an abnormal response of the body to these stress signals. In recent years, many studies have pointed out the body’s responses to external stress signals are regulated by miRNAs and aberrant miRNA regulation are intimately associated with the occurrence of the aforementioned conditions. The rapid responses of cells towards multiple stimuli in a complex environment are achieved and completed by signal transduction pathways. At the same time, miRNAs are used to achieve regulation [8, 11]. There are diverse miRNAs and they have many important physiological, biochemical, and gene regulatory functions. Each miRNA has many target genes and some miRNAs can regulate the same target gene. This results in the formation of a complex and intertwined signal regulatory network. One miRNA can regulate the expression of many target genes or the combination of several miRNAs can be used to fine-tune the regulation of some target genes [8, 11]. When stress occurs, miRNAs will use the following potential mechanisms for regulation: stress signal mediation, stress signal modulation, negative feedback and signal resolution, positive feedback and phenotypic switching, and buffering and signal stability. The activation of these 5 mechanisms differs but they have a common point in responses to stress signals, i.e., requiring corresponding miRNAs to achieve responses [8, 11, 15]. Once stress occurs, the corresponding miRNAs will be activated which will directly or indirectly regulate downstream signalling pathways to achieve responses to that stress signal [15]. A study has found that a deficiency in the corresponding miRNA molecule will result in an extremely slow response to that stress signal [15].

On the other hand, Endocytosis is a process in which extracellular substances are transported into cells through the deformation of plasma membranes [16–20]. According to the different size of the materials and the different mechanisms of endocytosis, it can be divided into three types, such as phagocytosis, pinocytosis and receptor mediated endocytosis [16–20]. Besides, synaptic vesicle cycle signal transduction pathway is a signal pathway involved in the process of chemical substance transmission in neuronal signal transduction [16–18, 21–25]. The vesicular vamp protein binds SNARE protein Syntaxin 1 and Snap25 on the neuron cell membranes, which leads the vesicle to fuse [16–18, 21–25]. The Munc18–1 protein combines monomeric Syntaxin 1 and SNARE complex and assists in the assembly of the complex [16–18, 21–25]. Complexin and Syt1 bind to SNARE protein to form a tight complex, which can aggregate lipid membranes. When the action potential in presynaptic neurons opens voltage-gated calcium channels, calcium ions combine with Syt1 and interact with SNARE complex and plasma membrane to fuse the membrane and release the electrical signals of neurons to the synaptic space [16–18, 21–25]. Above two signaling pathways cover intercellular material and signal transmission, which play an important role in cell survival, growth and normal physiological function. However, in the process of hemorrhoids, whether above signaling pathways regulate the infiltration of inflammatory cells, proliferation of vascular endothelial cells, and edema of interstitial cells and other abnormal pathophysiological processes remains to be further studied.

## Conclusions

In conclusion, this study indicates that the occurrence of haemorrhoids may be intimately associated with aberrant miRNA transcription, resulting in aberrant target gene expression and an imbalance in certain signal transduction pathways. In the future, functional studies will be conducted to define the relationship and underlying mechanism of these findings.

## Supplementary information

**Additional file 1: Figure S1.** Gene ontology analysis shows that haemorrhoids contain many physiological and biochemical abnormalities. (A–F) The target genes of upregulated miRNAs are mainly involved in biological regulation (BP), cell part (CC), and protein binding (MF). (G–L) The target genes of downregulated miRNAs are mainly involved in biological regulation (BP), cell part (CC), and protein binding (MF). **Figure S2.** Differentially expressed microRNAs (miRNAs) in haemorrhoids have many potential target genes. (A) Target gene prediction results showed that there are 184 potential target genes of significantly upregulated miRNAs (common to both TargetScan7.1 and MirdbV5 databases) and there are 372 potential target genes of significantly downregulated miRNAs (common to both TargetScan7.1 and MirdbV5 databases). (B) and (C) show corresponding potential target genes of significantly upregulated miRNAs. (D) and (E) show corresponding potential target genes of significantly downregulated miRNAs. **Figure S3.** Endocytosis signalling pathway. **Figure S4.** Synaptic vesicle cycle signalling pathway.

## Abbreviations

miRNA: microRNA
GO: Gene ontology
H&E: Haematoxylin-eosin
TPM: Tag counts per million miRNA alignments
MF: Molecular function
BP: Biological process
CC: Cellular component
