# Supplementary material for: Aberrant expression for microRNA is potential crucial factors of haemorrhoid
Source: Hereditas. 2020 Jul 3;157:25. doi: 10.1186/s41065-020-00139-9 (PMC7334851; doi:10.1186/s41065-020-00139-9)
Supplement: Supplementary file 1 — Additional file 1: Figure S1. Gene ontology analysis shows that haemorrhoids contain many physiological and biochemical abnormalities. (A–F) The target genes of upregulated miRNAs are mainly involved in biological regulation (BP), cell part (CC), and protein binding (MF). (G–L) The target genes of downregulated miRNAs are mainly involved in biological regulation (BP), cell part (CC), and protein binding (MF). Figure S2. Differentially expressed microRNAs (miRNAs) in haemorrhoids have many potential target genes. (A) Target gene prediction results showed that there are 184 potential target genes of significantly upregulated miRNAs (common to both TargetScan7.1 and MirdbV5 databases) and there are 372 potential target genes of significantly downregulated miRNAs (common to both TargetScan7.1 and MirdbV5 databases). (B) and (C) show corresponding potential target genes of significantly upregulated miRNAs. (D) and (E) show corresponding potential target genes of significantly downregulated miRNAs. Figure S3. Endocytosis signalling pathway. Figure S4. Synaptic vesicle cycle signalling pathway. [file 41065_2020_139_MOESM1_ESM.pdf]

Aberrant expression for microRNA is potential crucial factors of haemorrhoid

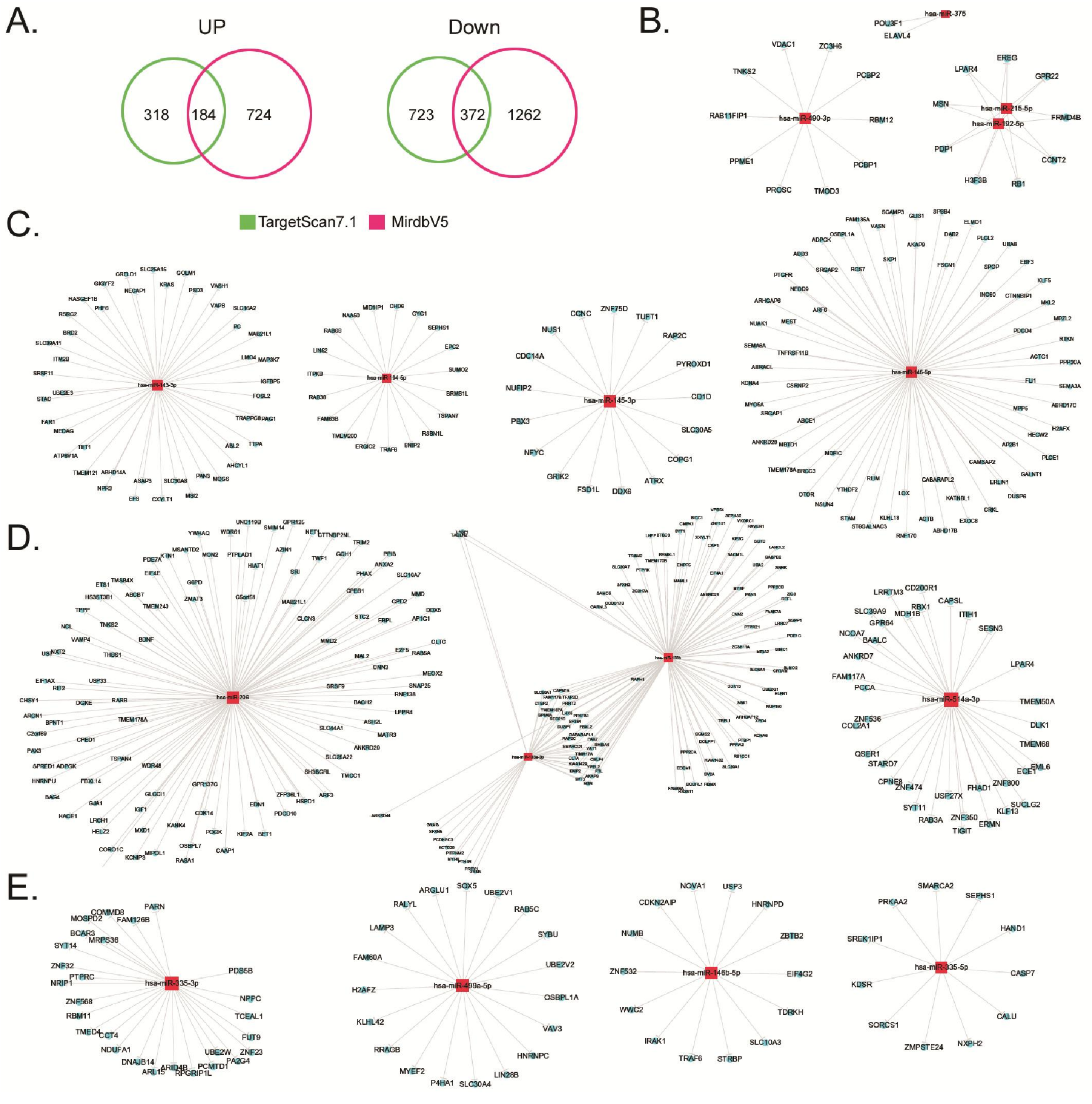

Figure S1. Gene ontology analysis shows that haemorrhoids contain many physiological and biochemical abnormalities. (A–F) The target genes of upregulated miRNAs are mainly involved in biological regulation (BP), cell part (CC), and protein binding (MF). (G–L) The target genes of downregulated miRNAs are mainly involved in biological regulation (BP), cell part (CC), and protein binding (MF).

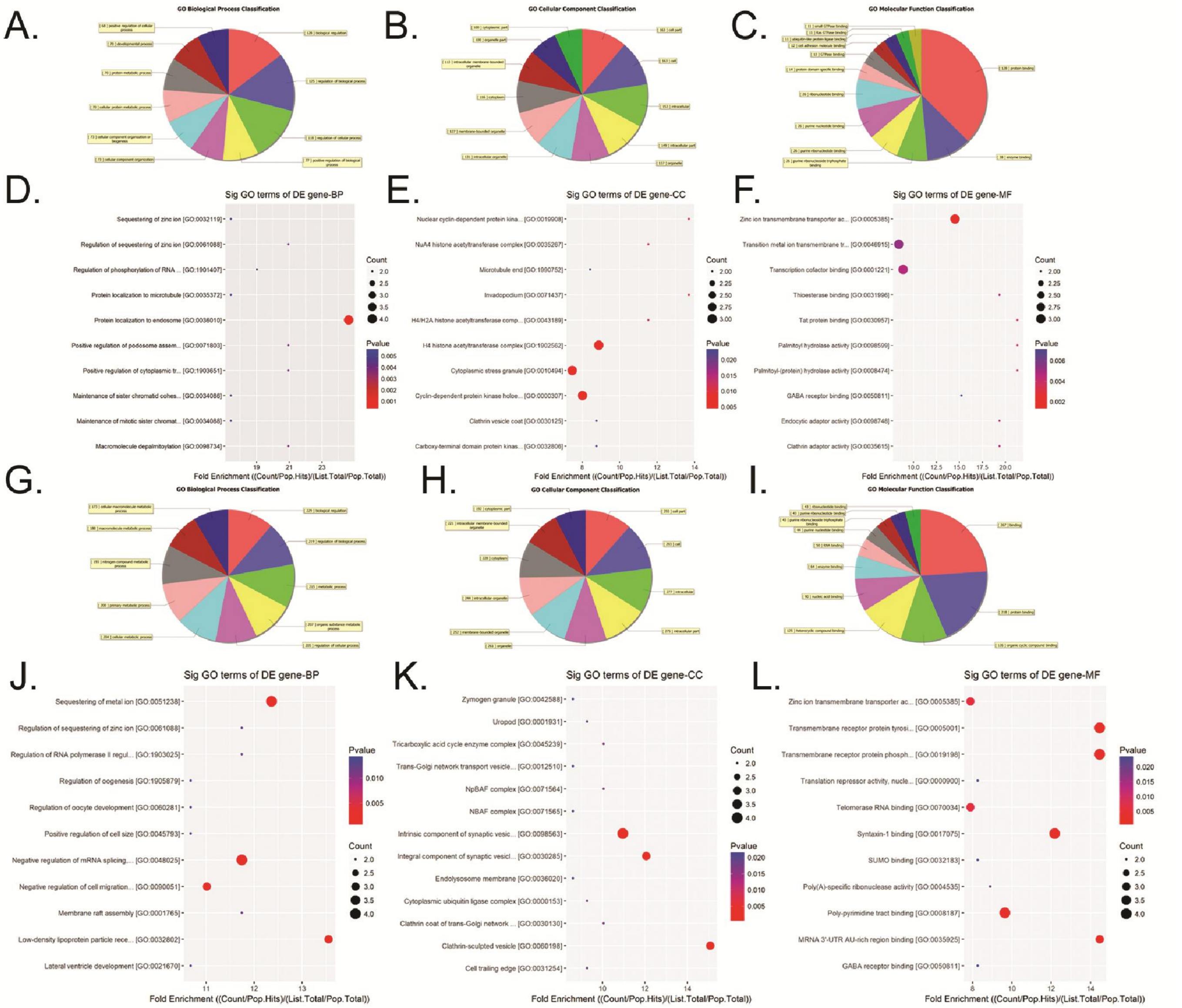

Figure S2. Differentially expressed microRNAs (miRNAs) in haemorrhoids have many potential target genes. (A) Target gene prediction results showed that there are 184 potential target genes of significantly upregulated miRNAs (common to both TargetScan7.1 and MirdbV5 databases) and there are 372 potential target genes of significantly downregulated miRNAs (common to both TargetScan7.1 and MirdbV5 databases). (B) and (C) show corresponding potential target genes of significantly upregulated miRNAs. (D) and (E) show corresponding potential target genes of significantly downregulated miRNAs.

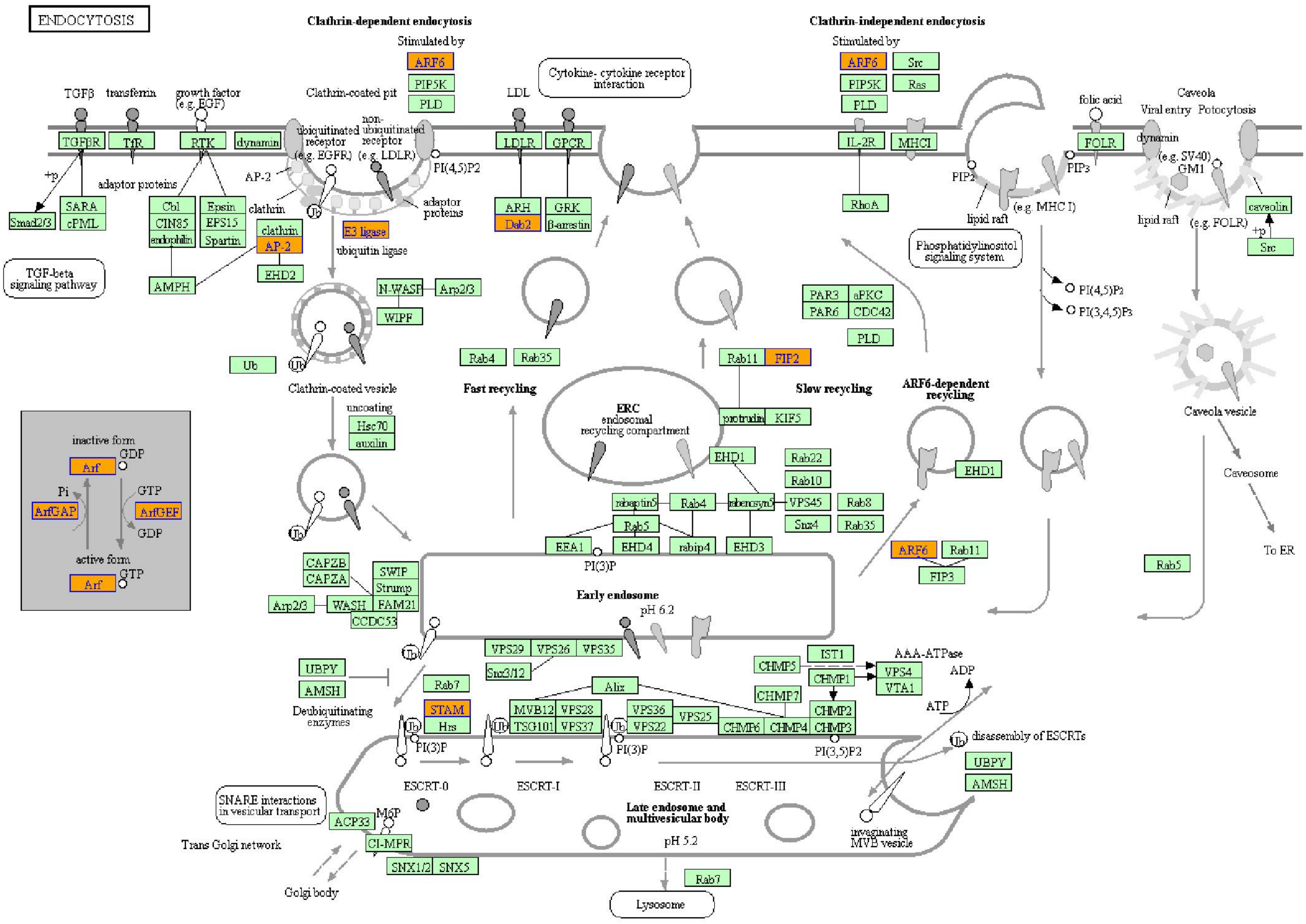

Figure S3. Endocytosis signalling pathway.

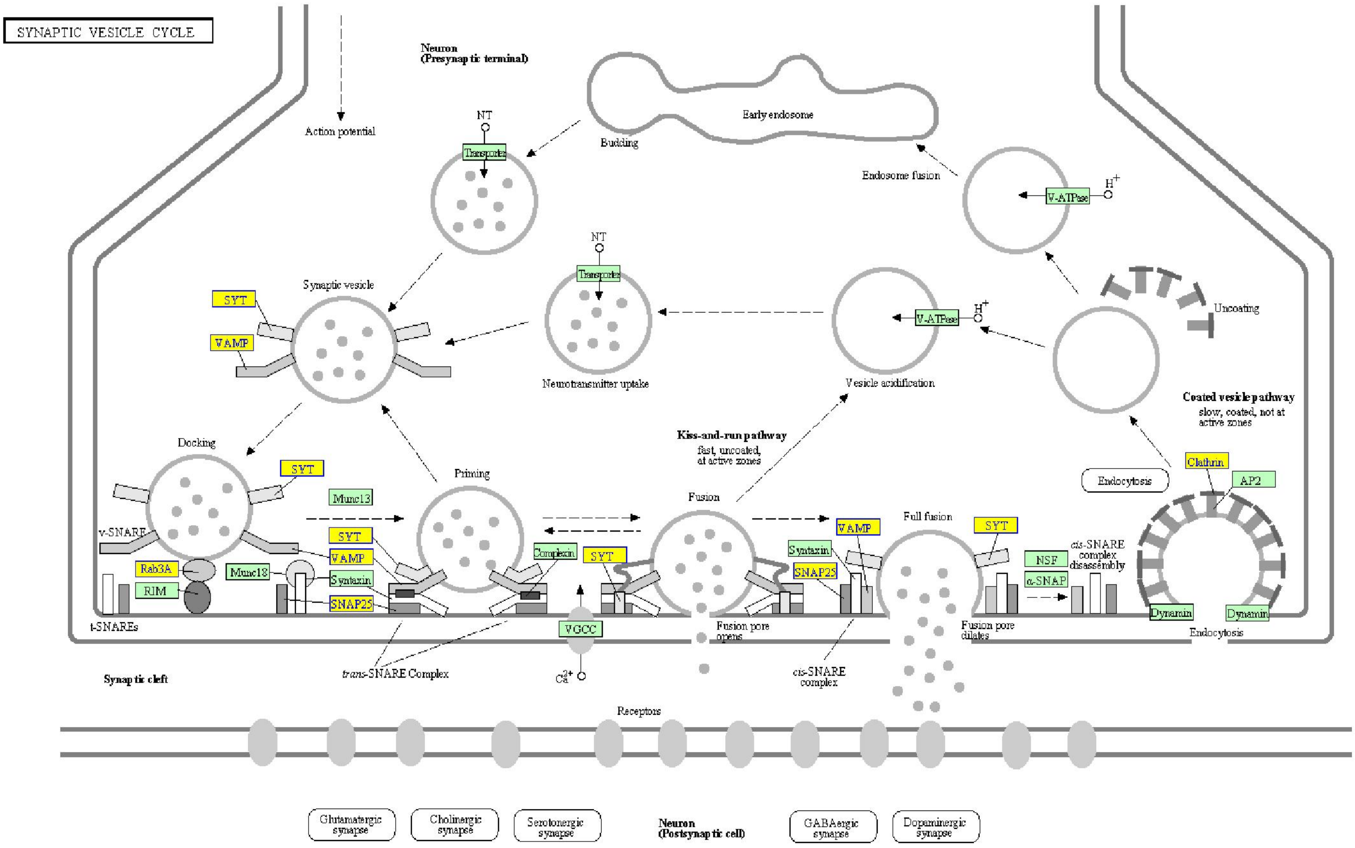

Figure S4. Synaptic vesicle cycle signalling pathway.
